# New Insights into RPE-Photoreceptor Complex Ultrastructure using Focused Ion Beam-Scanning Election Microscopy (FIB-SEM)

**DOI:** 10.21203/rs.3.rs-3200741/v1

**Published:** 2023-08-07

**Authors:** Shermaine W.Y. Low, Rayne R. Lim, DeAna G. Grant, Sam Patterson, Shyam S. Chaurasia

**Affiliations:** Medical College of Wisconsin; University of Washington; University of Missouri; Medical College of Wisconsin; Medical College of Wisconsin

**Keywords:** Retina, Photoreceptors, Retina pigment epithelium, Interdigitation Zone, Transmission Electron Microscopy, Focused Ion Beam Scanning Electron Microscopy

## Abstract

Photoreceptors in the retina are specialized neuronal cells that perceive light and play a central role in the visual system. Damage to photoreceptors is a clinical feature often associated with various retinal degenerative disorders. The photoreceptor bed comprises a unique extracellular matrix (ECM) scaffold often described as the interphotoreceptor matrix (IPM) in the subretinal space, vital during retinal development and homeostasis. In this study, we used focused ion beam scanning electron microscopy (FIB-SEM) and transmission electron microscopy (TEM) to analyze the ultrastructural architecture of the retinal pigmented epithelium (RPE)-photoreceptor complex in mice. Additionally, we describe methods for retinal preparation in EM imaging. TEM images display ultrastructural retina layers, including Bruch’s membrane and the interdigitation zone (IZ). The 3-dimensional reconstruction of the outer retina revealed individual photoreceptors, the connection between their inner and outer segment via the photoreceptor cilia, and photoreceptor interaction with the RPE ciliary processes. Our findings highlight the importance of FIB-SEM in deciphering the ultrastructural details of RPE-photoreceptor interactions in the IPM complex which are essential for the maintenance of retinal architecture.

## Introduction

The retina is a neural tissue located in the posterior portion of the eye comprising intrinsic components required for the vision system. First, the photoreceptors (rods and cones) process light entering the eye into graded potentials. Then, it sends them to the visual cortex in the brain for three-dimensional (3-D) vision processing ^1^. Rod and cone photoreceptors are inherent cells that act as the first responders to light ^2^. Rods are sensitive to dim light and are responsible for vision in scotopic conditions, while cones are responsible for high photopic visual acuity ^3^. These photoreceptors have a distinct inner segment containing mitochondria and ribosomes responsible for opsin production. Opsins are then passed to the light-sensitive outer segment of the photoreceptors, where they arrange into stacked discs. The rod outer segments are generally longer than cone outer segments and comprise individual discs surrounded by the ciliary plasma membrane, isolating them from the extracellular space. Cone outer segments, in contrast, arise from evaginations and invaginations of the ciliary plasma membrane and are exposed to the extracellular matrix (ECM) to facilitate rapid phototransduction required for high photopic resolution ^4^.

The retinal pigment epithelium (RPE) is a specialized retinal layer between the choroid and the neural retina. It has numerous functions, including nutrient transport, protection against photooxidation, maintenance of retinal structural integrity, and phagocytosis of discarded photoreceptor membranes ^5^. Photoreceptors constantly renew their outer segments via shedding to maintain their excitability. Newly processed photoreceptor outer segments (PhR-OS) develop from the cilium and push photodamaged disk segments towards the distal tips in a process often described as disc shedding. RPE works with the photoreceptors by removing the shed tip via phagocytosis, forming an interdigitation zone (IZ) where the apical processes of RPE microvilli ensheaths the PhR-OS ^6^. Damage to these photoreceptors and the IZ can thus result in various retinal degenerative disorders, including retinitis pigmentosa (RP), cone dystrophy, cone-rod dystrophy, Usher’s syndrome, Lebel congenital amaurosis and color blindness ^7,8^. Furthermore, these photoreceptor cells have been reported to play an important role in the development of retinal vascular disorders such as in diabetic retinopathy (DR) ^9^. Neurodegeneration can also result in downstream vascular defects which is a characteristic of DR ^10^.

In the retina, photoreceptors depend on the interphotoreceptor matrix (IPM) for proper physiological development ^11^. The IPM is a unique extracellular matrix (ECM) that occupies the subretinal space in the outer retina surrounding photoreceptors and the RPE. Various proteins and carbohydrates comprise the retinal IPM, forming an organized meshwork surrounding retinal cells.

Electron microscopy is a common tool for evaluating biological ultrastructure at the nanometer scale. Transmission electron microscopy (TEM) is widely available for analyzing biological samples by engaging an electric current to heat a tungsten filament to high voltages required for generating a fine electron beam. The beam passes through ultrathin sections of samples pre-treated with heavy metals such as uranyl or lead which impedes the electron beam in proportion to their electron density. These differences in the transmission of the emitted electrons are then used to create a high-resolution cross-sectional image that provides valuable information on tissue morphology. On the other hand, scanning electron microscope (SEM) uses a beam of high-energy electrons that strikes sample surfaces to produce secondary and backscattered electron signals. The sample generates secondary electrons due to low energy and inelastic interactions with the beam and is primarily used for topographic inspection. In contrast, backscattered imaging results from incident electrons deflected away from “heavier elements,” providing crystalline and magnetic field information ^12,13^. A detector captures these signals to produce topographical and morphological data to visualize cellular structures. The rapid speed and large field of view in the SEM give it a considerable advantage over TEM when studying the morphology of biological tissues ^14^. Despite the prevalence of its use for biological specimens, these EM methods come with their caveats. Some major disadvantages of using TEM or SEM include laborious preparation of samples, a requirement for specialized equipment, and artifact formation from inexperienced handling of samples. While ultrathin sections are essential for TEM to work, SEM samples cannot show the specimen’s internal structures, and both techniques only produce 2-D micrographs, in greyscale.

In recent years, SEM has been coupled with focused ion beam (FIB) technology for the 3-D reconstruction of biological tissues ^15,16^. After each SEM image is captured, FIB-SEM uses a focused gallium ion beam to remove a thin layer of the tissue before the next image is captured. Collected images are then stacked and computationally segmented to allow 3-D visualization of the tissue. This method reveals the morphological features of the structures of interest and uncovers spatial relationships between tissue ultrastructure that would otherwise be incomprehensible in standard 2-D SEM imaging ^14^. The automated process of sectioning and imaging also allows for quicker data collection than traditional serial TEM. Additionally, the nanoscale physical sections milled by the FIB allow for high-resolution data capture ^17^. In the present study, we sought to determine the ultrastructural architecture of the RPE-photoreceptor junction in the retina. Understanding the structural architecture of individual photoreceptors and their interactions with their surrounding RPE cells may provide valuable insights into their roles in signal transmission or the maintenance of retinal structural integrity. Additionally, deciphering the structural components that enable proper retinal function will be crucial for developing new therapeutic strategies to combat photoreceptor loss or degenerative diseases in the retina. However, evaluating these structurally complex retinal neural networks requires imaging techniques with extremely high resolution. Therefore, to further understand the ultrastructural changes in the RPE-photoreceptor complex during retinal disease, we describe the methodology for preparing and examining using FIB-SEM technology.

## Materials and Methods

### Posterior eye cup preparation

All experimental animal protocols were approved by the Institutional Animal Care and Use Committee (IACUC) of the University of Missouri, Columbia, MO. The study was conducted in accordance with the ARRIVE guidelines and with the principles for the Use of Animals in Ophthalmic and Vision Research by the Association of Research for Vision and Ophthalmology (ARVO). C57BL6/J mice were obtained from The Jackson Laboratory. Mice were euthanized at ages eight to twelve weeks old via cervical dislocation and entire eye globes enucleated. Samples were prepared following a modified version of the National Center for Microscopy and Imaging Research (NCMIR) methods for 3-D EM. Unless otherwise stated, all reagents were purchased from Electron Microscopy Sciences, and all specimen preparation was performed at the Electron Microscopy Core Facility, University of Missouri. Tissues were fixed in 2% paraformaldehyde and 2% glutaraldehyde in 100 mM sodium cacodylate buffer pH 7.35. After 10 minutes, the globes were moved onto a clean petri dish with a fresh fixative solution for dissection. The anterior tissues of the eye, including the cornea, ciliary body, and lens, were carefully removed to prevent retinal detachment. HistoGel (ThermoFisher Scientific, Waltham, MA, USA) was then pipetted directly into the eye cup and allowed to solidify to immobilize the posterior tissue. The eye cup in HistoGel is resubmerged into electron microscopy fixative and kept at 4°C until further processing.

### Processing for electron microscopy

Fixed tissues were rinsed with 100 mM sodium cacodylate buffer (pH 7.35) containing 130mM sucrose. Secondary fixation was done with equal parts 4% osmium tetroxide and 3% potassium ferrocyanide in cacodylate buffer, incubated on ice for 1 hour, then rinsed with cacodylate buffer and further with distilled water. En block staining was performed for 1 hour in a 1% thiocarbohydrazide solution, followed by distilled water rinses. Tissues were then incubated in an additional 2% aqueous osmium tetroxide solution for 30 minutes at room temperature, then rinsed with distilled water. Further en bloc staining was performed using 1% aqueous uranyl acetate, incubated at 4°C overnight, and then rinsed with distilled water. A final en bloc staining was performed using Walton’s Lead Nitrate solution for 30 minutes at 60°C. Next, tissues were rinsed and dehydrated using ethanol, transitioned into acetone, infiltrated with Durcapan resin, and polymerized at 60°C overnight. Retinal eyecups were halved transversely to reach the center of the retina near the optic nerve. The tissue block was then trimmed to 500µm length, and block faces were prepared using an ultramicrotome (Ultracut UCT, Leica Microsystems, Germany) and a diamond knife (Diatome, Hatfield, PA, USA). Samples were mounted on an SEM stub and coated with 20nm platinum using the EMS 150T-ES Sputter Coater. To check tissue orientation and establish landmarks prior to FIB-SEM, 75nm sections were obtained for TEM. Images were acquired with a JEOL JEM 1400 transmission electron microscope at 80 kV on a Gatan Ultrascan 1000 CCD at 1200X magnification.

### Focused Ion Beam-Scanning Electron Microscopy (FIB-SEM)

The tissue block was mounted on an SEM stub and coated with 20nm platinum using the EMS 150T-ES Sputter Coater. Serial block face (SBF) data were acquired on a ThermoFisher Scientific Scios Analytical Dualbeam (Hillsboro, OR). First, the region of interest was identified using established landmarks and protected with a 1µm layer of platinum using the ion column. Next, trenches were rough cut to the block face’s side and front using a high ion beam current (30kV 5nA) to expose the desired block face. Next, the block face was polished using an ion beam current of 50pA prior to collecting serial images using the Slice & View automated software package. Serial sections were cut at a thickness of 20nm (30kV 1nA), and SEM images were acquired at 2keV and 25pA using the T1-BSE detector and reverse contrast.

### Image Segmentation

**Figure 1:**
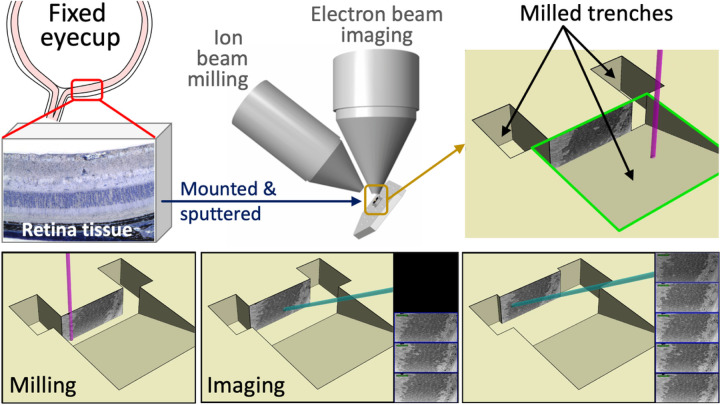
Schematic of mouse retinal sample preparation for FIB-SEM imaging. Mouse eyes were enucleated and fixed in electron microscopy fixative. The anterior chamber including the cornea, ciliary body and lens was then dissected and removed. Posterior eye cups were then submerged in HistoGel. Retinal samples were mounted, and sputter coated onto a SEM. Samples were then milled and imaged using FIB-SEM technology. Captured images were stacked to form the 3-D reconstruction of photoreceptor layers.

Image segmentation was performed using ThermoFisher Scientific Amira 6 Software. An area with intact and complete serial images of photoreceptors is selected from each sample. Then, the RPE, photoreceptor outer segments, and inner segments within the area are manually segmented to obtain a 3-D reconstruction of the cells. A schematic for mouse retinal sample preparation and FIB-SEM processing is represented in Fig. 1. Video representation for FIB-SEM processing can be found in Supplementary Video S1.

## Results

### TEM analysis shows retinal pigmented epithelium, interdigitation zone, and photoreceptor distribution

**Figure 2:**
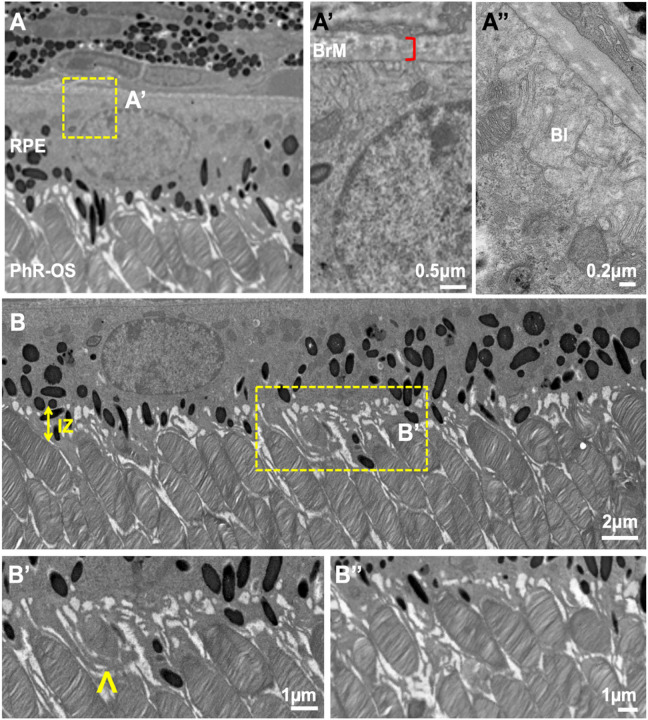
TEM images of wild type mouse eyes. TEM images of wild type mouse eyes were captured using a JEOL JEM 1400 microscope at 80 kV on a Gatan Ultrascan 1000 CCD at 1200X magnification. Images were further enlarged for analysis of wild type mice retinal layers. The RPE was polarized with apical microvilli and BI (A’’) adjacent to the BrM (A’). Electron dense pigment granules are distributed in the middle and apical portions of the RPE, with some located in the apical processes. IZ is uniformly spaced throughout the retina (B). PhR-OS were regularly spaced across the RPE, and apical processes were seen interdigitating with individual PhR-OS (B, B’’). PhR disc are engulfed by microvilli in preparation for phagocytosis into the RPE (B’, arrow). TEM, Transmission Electron Microscopy; RPE, Retinal Pigmented Epithelium; BI, basal infolding; BrM, Bruch’s Membrane; IZ, Interdigitation zone; PhR-OS, Photoreceptor Outer Segment.

Mice retinal samples were captured using a JEOL JEM 1400 TEM at 80 kV on a Gatan Ultrascan 1000 CCD using 1200X magnification (Fig. 2). We found significant details of the retinal architecture in the tissue, including the Bruch’s membrane (BrM) and the IZ. The RPE was polarized with apical microvilli facing inwards to the photoreceptor and basal infoldings (Fig. 2A’’, BI) on the basal side adjacent to the BrM (Fig. 2A’, bracket). The BrM in RPE can be easily identified, with uniform thickness along the entire RPE (Fig. 2A’). Electron-dense pigment granules are distributed predominantly on the apical side of the RPE, with some located within the microvilli. In contrast, RPE mitochondria were localized towards the basal third of the cell, consistent with observations made in human RPE ^18^. PhR-OS were consistently aligned and evenly spaced throughout the imaged field (Fig. 2B). IZ thickness varies slightly across the RPE monolayer. However, the RPE apical processes were seen to interdigitate every outer segment, with some engulfing retinal PhR-OS in preparation for phagocytosis (Fig. 2B’, arrow).

#### FIB-SEM allows for the analysis of photoreceptors connecting cilia and mouse interdigitation of the photoreceptor outer segments with RPE microvilli.

**Figure 3:**
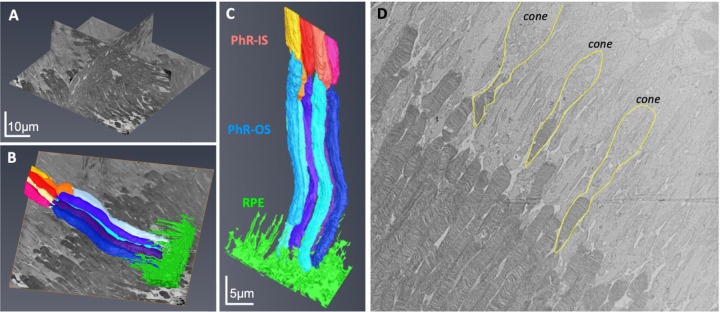
Photoreceptors in wild type mice. Orthogonal view of the milled block along the different axes (A). Individual rods and the RPE were 3-D reconstructed and digitally colored (B, C). Majority of photoreceptors were rods, with the cones (outlined in yellow) easily identified by a wider PhR-IS and accumulation of mitochondria in the center of the IS (D). PhR-IS, Photoreceptor Inner Segment; PhR-OS, Photoreceptor Outer Segment; RPE, Retinal Pigmented Epithelium.

**Figure 4:**
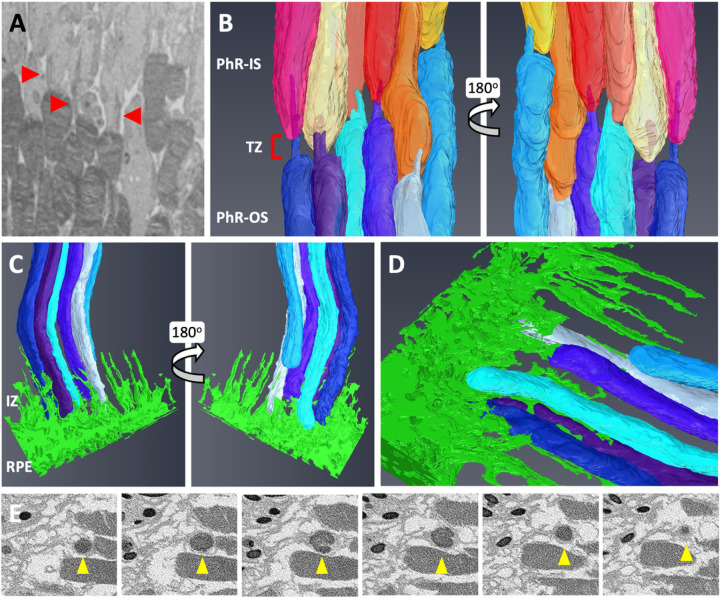
Segmentation revealed interconnecting cilium and interdigitation zone in the RPE-photoreceptor complex. The cilium in rods (red arrowhead) was identified and digitally segmented as part of the PhR-OS (A, B). Digital reconstruction revealed the cilium and TZ in every rod (B). At the RPE-photoreceptor complex, RPE apical processes extend upwards to the PhR-OS distal tips, forming the IZ (C, D). Sequential image slices taken at the distal tip demonstrate the engulfment of a shed disc (yellow arrowhead) by the RPE microvilli (E). RPE, Retinal Pigmented Epithelium; PhR-OS, Photoreceptor Outer Segment; PhR-IS, Photoreceptor Inner Segment; TZ, Transition Zone; IZ, interdigitation Zone.

After analysis of TEM images, an area is selected for further FIB-SEM examination. Orthogonal views of the complete milled block are represented in Fig. 3A. The X, Y and Z-axis dimensions for the segmented areas were 45µm, 45µm and 12µm, respectively. Six complete rods in the middle of the block with distal interactions with the RPE were isolated for segmentation. Colors were assigned to individual rod inner and rod outer segments (ROS) to identify ciliary interactions, and RPE was colored as a block to visualize the IZ (Fig. 3B). The ROS volume was approximately 77.8µm^3^ ± 9.6µm^3^ (Fig. 3C), more prominent than the dimensions previously reported ^19,20^. The differences could be attributed to the location of the rod in the retina, and the time of collection since ROS lengths were shown to change over 24h ^21^. Most photoreceptors in the murine retina were rods, with cones organized among the rods ^20^ (Fig. 3D). The inner and outer segments in both rods and cones are connected by a cilium, easily identified in an image slice (Fig. 4A). Using FIB-SEM, we could digitally reconstruct every rod’s cilium and transition zone (TZ), which would otherwise be missed in traditional 2-D EM (Fig. 4B; Supplementary Video S2). The RPE (green block) has numerous apical microvilli extending into the photoreceptor space (Fig. 4C; Supplementary Video S3). The photoreceptors were nestled between the RPE apical processes forming close interactions with the elongated microvilli (Fig. 4D). Sequential image slices at the IZ revealed the encapsulation of a shed disc by the RPE microvilli (Fig. 4E), which would be phagocytosed by the RPE eventually.

## Discussion

Photoreceptors are critically important neuronal cells in the retina that mediate visual function by transmitting visual information for image processing in the brain. Damaged photoreceptors can result in various downstream signal transmission defects and produce numerous retinal disorders, including RP, Usher’s syndrome, and DR ^7,9^. Thus, photoreceptor structure and function are crucial for visual capabilities. Due to the micrometer scale of retinal cellular structures, high-resolution techniques are required for detailed analysis of retinal architecture. To understand the physiological layout and morphology of retinal photoreceptors, we used TEM and FIB-SEM to examine the fine ultrastructure of the mouse retina.

TEM technology employs a high-voltage electron beam that scatters electrons across the surface of a sample and collects their resultant distribution using a fluorescent screen on the bottom of the microscope ^22^. Performed on a single sample, it generates a purely 2-D image. Ultrathin TEM sections can then be employed to reconstruct 3-D tissue segments. However, this approach is time-consuming and technically demanding. Additionally, structures parallel to the 2-D plane are easily lost ^23^. As such, FIB-SEM analysis was subsequently selected to further investigate the mouse retina. The principal advantages of FIB-SEM include its high-resolution imaging of diverse biological tissue, 3-D ultrastructure, isotropic data, and relatively fast processing speed. The ability to simultaneously image intracellular structures and gross morphology, along with the automated process of tissue reconstruction, significantly reduces image processing time ^24^. Furthermore, the ease of reorienting the isotropic 3-D data set in FIB-SEM allows for a high-resolution examination of arbitrary slices ^23^. Studies have also found that FIB-SEM imaging was more sensitive for detecting synaptic connections than TEM ^25^. FIB-SEM also allows for the complete reconstruction of a photoreceptor, regardless of the sectioning angle, which is incredibly challenging to predict for 2-D TEM.

**Figure 5:**
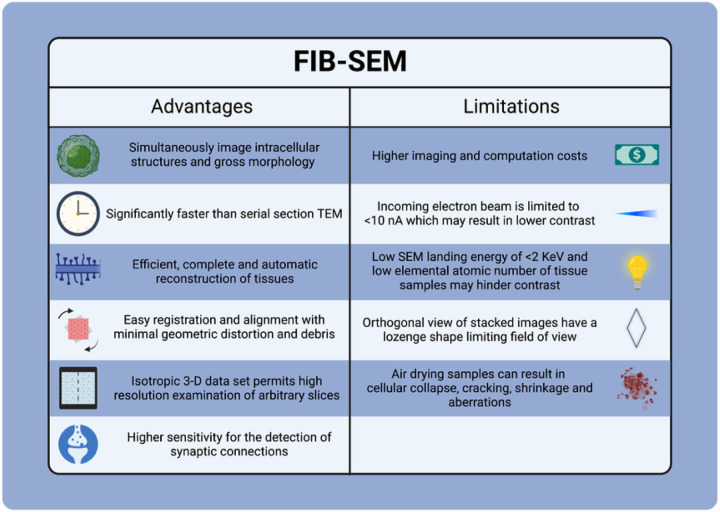
Summary of the advantages and limitations of FIB-SEM imaging.

However, as with many considerable technological advances, FIB-SEM imaging has limitations. Despite the clarity of biological tissue images collected by FIB-SEM, the imaging process remains imperfect and is limited by the bottleneck of image segmentation. For one, most elements in biological specimens are of relatively low atomic number, resulting in irregular contrast across the image ^24^. In addition, the delicate interaction between the beam of electrons and the sample elements require an incoming electron beam of less than 10 nA. Using a higher SEM current beam would requires a larger aperture subject to Coulomb repulsion, creating chromatic aberration and blur ^23^. Low SEM landing energy (< 2 keV) used to reduce electron penetration depth can also reduce image contrast ^26^. Furthermore, the orthogonal view of the aligned and stacked image volume often has the shape of a lozenge due to the typical orientation of the SEM column, limiting the field of view ^26^. Air-drying samples can also lead to unfavorable cellular aberrations ^27^. A list of advantages and limitations of using FIB-SEM have been summarised in Fig. 5.

The BrM is a thin, penta-laminar structure that strategically separates the retina from the choroid and acts as a filter for nutrient, ion, and fluid exchange between the retina and the systemic circulation. It also plays a structural role in the eye by stretching and returning to its original form according to changes in intraocular pressure and choroidal blood volume ^28,29^. Furthermore, drusen formation in between the RPE-basal lamina and the inner collagenous zone changes BrM thickness and is associated with several devastating retinal diseases, including RP, DR, and age-related macular degeneration (AMD) ^29^. TEM micrographs of mouse eyes provided clear delineation layers of the BrM (Fig. 2A’), allowing for retinal layer analysis and thickness measurements. Further application using the FIB-SEM technique could also provide useful 3-D information for Drusen analysis.

FIB-SEM imaging also accurately reconstructed the connecting cilium along the transition zone connecting the inner and outer segments of photoreceptors (Fig. 4A-B; Supplementary Video S2). This is often missed in 2-D TEM, which depends on the sectioning angle. Digital reconstruction of photoreceptor and their cilium in wild type vs. animal models of ciliopathies could uncover valuable information regarding the progression of degeneration. Meta analysis from digital segmentation will also allow for comparing the length and volume of each photoreceptor. This FIB-SEM dataset also uncovered precise interactions between the PhR-OS and the extended apical processes of the RPE (Fig. 4C-D; Supplementary Video S3). Since ensheathment for the outer segment tips play a role in the retinal attachment ^5,30^, disruption to this interaction can result in photoreceptor degeneration, retinal detachment, and eventual impairment in vision.

Most importantly, FIB-SEM provided high-resolution micrographs of retinal ultrastructure revealing valuable information for IZ analysis. We found the mouse retina had regularly spaced IZ and uniformly distributed photoreceptors (Fig. 2B). Disruption to the RPE microvilli, which which unsheathes the PhR-OS, can result in the loss of IZ integrity and lead to an absence in IZ bands when observed under OCT. Changes in IZ thickness observed using optical coherence tomography (OCT) imaging have been associated with several retinal disorders, including glaucoma, diabetic retinopathy (DR), diabetic macular edema (DME), retinitis pigmentosa (RP), and retinopathy of prematurity (ROP) ^31–34^. Loss of IZ bands has also been used to predict visual acuity outcomes post-retinal surgeries and used as a marker for photoreceptor damage ^35,36^. While several animal models for the abovementioned retinal disorders are commonly used, achieving a clear IZ band using OCT imaging may be difficult for small animal models. The method described in this paper provides enlarged and high-resolution micrographs of the IZ, which may highlight inappreciable differences using former techniques and provide vital information for detecting abnormalities due to changes in the IPM and its effects on the RPE-photoreceptor complex.

## Conclusions

We report new insights into retinal 3-D ultrastructural interactions between RPE apical processes and PhR-OS using state-of-the art FIB-SEM technology. We described the process for tissue sample preparation, image segmentation, and 3-D reconstruction of the mouse’s outer retina. The advantages and caveats of using FIB-SEM technology were also discussed.

## Supplementary Material

Supplement 1
