# Supplementary material for: New Insights into RPE-Photoreceptor Complex Ultrastructure using Focused Ion Beam-Scanning Election Microscopy (FIB-SEM)
Source: Res Sq. 2023 Aug 7:rs.3.rs-3200741. Preprint. [Version 1] doi: 10.21203/rs.3.rs-3200741/v1 (PMC10441459; doi:10.21203/rs.3.rs-3200741/v1)
Supplement: Supplement 1 [file NIHPPRS3200741v1-supplement-1.pdf]

Supplementary Files

This is a list of supplementary files associated with this preprint. Click to download.

- SupplementaryVideoS1Chaurasia.m4v
- SupplementaryVideoS2Chaurasia.mpg
- SupplementaryVideoS3Chaurasia.mpg
